# Effects of the portage early education program on Chinese children with global developmental delay

**DOI:** 10.1097/MD.0000000000012202

**Published:** 2018-10-12

**Authors:** Xiumei Liu, Xue-Ming Wang, Jing-Jing Ge, Xiu-Qing Dong

**Affiliations:** Department of Pediatrics, Yuhuangding Hospital of Qingdao University, Yantai, Shandong, P.R. China.

**Keywords:** developmental quotient, early intervention, global developmental delay, portage guide to early education

## Abstract

Children with global developmental delay (GDD) were trained with the Portage Guide to Early Education (PGEE) program.

In the treatment group, the PGEE program was performed on children with GDD (45 cases) through a combination of family and hospital interventions, in a 1-to-1 ratio. The Gesell Infant Development Scale (GESELL) developmental quotient (DQ) and social adaptability were measured before and 6 months after PGEE implementation in the treatment group. These parameters were also evaluated in a control group (30 cases) during an initial visit and 6 months later.

Before the PGEE intervention, no significant differences were observed between the general characteristics of children in the control and treatment groups. Six months after the PGEE intervention, the DQ values of the children with GDD in the treatment group (64.7 ± 9.5) were significantly higher than those before treatment (54.6 ± 9.3) and those of the control group (58.3 ± 10.2) (*P* < .05). The PGEE intervention significantly increased the DQ values on 5 aspects, including gross motor, fine motor, adaptability, language, and personal social activity abilities, and the scores on the Infants-Junior Middle School Students’ Social-Life Abilities Scales (SM scales), as compared with the control group (*P* < .05).

The PGEE program improves the DQ, social adaptability, and prognosis of children with GDD.

## 1 Introduction

Developmental delays (DDs) are common childhood health problems that lead to limitations in 3 or more activities of life, including selfcare, acceptance and expression, language learning, activities, self-guidance, independent living, and economic self-sufficiency. DD can be classified as global developmental delay (GDD), Down syndrome, mental retardation, developmental language disorder, motor delay (MD), cerebral palsy, autistic spectrum disorder (ASD)/pervasive DD, and special sensory dysfunction (e.g., blindness, deafness). Evidently, DD is not a very accurate name for diagnostic purposes; however, it is used widely in clinical practice.

GDD is a common chronic neurodevelopmental disorder in infants and children aged less than 5 years, and it affects 1% to 3% of the population. It is defined as a significant delay in 2 or more domains of development, including gross/fine motor skills, cognition, speech/language, personal/ social skills, or activities of daily living.^[1,2]^ It is common among children, and it is characterized by varied clinical manifestations. Some children with GDD also have physical malformations (small/large head circumference, high palatal arch, low auricular position, etc) or visceral malformations (heart, kidney, liver, or central nervous system malformations). During infancy and early childhood, the clinical manifestation of GDD includes language retardation (lack of vocabulary, inarticulate, poor comprehension, and limited in usage of vocabulary and grammar as compared with normal children of the same age) and motor retardation (the starting age for actions such as rising prone, sitting, standing, walking, and other actions are later than those for normal children). In the meantime, children with GDD often have low social living ability, and they exhibit an insufficient understanding of and adaptation to the surrounding environment. The development outcomes of GDD vary significantly across children. The outcome of DD affects the quality of life of children with GDD, and severe cases can cause permanent disability.^[3,4]^ Early intervention in children with GDD can help some children recover from this disorder. For instance, timely and effective early intervention can avoid lacks in intelligence quotient (IQ) in children with GDD. On the contrary, those who do not receive such timely intervention, or those with severe DDs may develop DD. Therefore, early and effective intervention is needed to improve the quality of life of children with GDD.

Early intervention is a comprehensive service provided for children with DD or for those at a high risk of DD, to improve their cognition, emotion, behavior, and ability to adapt to the society. At present, many countries in the world are exploring systems to manage early intervention. In the United States, most early interventions are home-based services. The most important part of intervention is the collaboration and consultation with the family. In China, GDD in children is regarded as an important public health and social problem, and rehabilitation is the most commonly used forms of early intervention. Rehabilitation includes institution-based programs such as physical therapy and occupational therapy, which are provided in clinics. Therefore, parents have to bring their children with GDD to the institution to receive treatment. It is inconvenient and expensive for children with GDD and their families to avail such services. Considering this situation, it is necessary to improve the implementation and management of early intervention and make it more suitable for Chinese children. Institution- and home-based services should be combined in the early intervention for children with GDD.

Early childhood intervention has been shown to lead to a positive outcome in children with DD.^[4]^ Early intervention advocates the provision of services in the natural environment. Usually, children's growing environment of learning and entertainment is considered as their natural environment, including their family, childcare center, and the community. The most common natural environment is the family. The Portage Guide to Early Education (PGEE) program can be conducted in families, communities, rehabilitation institutions, and early education centers, but the role of the family and parents needs to be highlighted. The PGEE program has been used widely for early intervention in children with GDD due to its scientific, interesting, coherent, and operable nature.^[5]^ It is an early intervention method for cognitive training, which can be used as structured teaching arrangements. However, the core status of game activities, toys, books and stories, and daily life should be emphasized.

Most of the studies in the literature are about children with ASD or cerebral palsy. Studies on the outcomes of children with GDD are surprisingly few.^[11]^ In the present study, we applied the PGEE program to children with GDD through a combination of institution- and home-based services and observed the resulting effects.

## 2 Methods

### 2.1 Participants

Children were recruited from the Children's Health Clinic in Yantai Yuhuangding Hospital. The program was implemented from January 2014 to January 2016. After evaluation by a developmental behavioral pediatrician based on the guidelines of the American Academy of Neurology, children who presented all of the following characteristics were included in our study: DD in 2 or more domains, age under 5 years, and too young to be assessed with a standardized intelligence test.

Children with the following characteristics were excluded from our study: children with GDD who presented severe hearing disorders, children with GDD who were also diagnosed with hereditary metabolic and chromosomal diseases, children diagnosed with neurodevelopmental disorders, including cerebral palsy, developmental coordination disorders, autism spectrum disorders, and neuromusculoskeletal disorders such as muscular dystrophy, children with GDD who had participated in an early intervention or rehabilitation program previously, parents and children who could not attend the PGEE program once per week, and patients who did not complete at least 6 months of the PGEE program.

**Figure 1:**
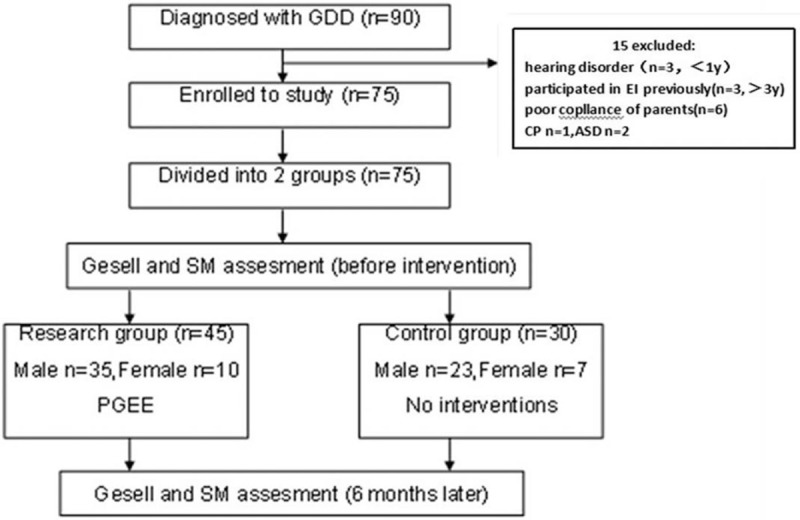
Research design and flow. ASD = autism spectrum disorders, CP = cerebral palsy, EI = early intervention, GDD = global developmental delay, Gesell = Gesell Infant Development Scale, PGEE = Portage Guide to Early Education, SM = Infants-Junior Middle School Students’ Social-Life Abilities Scales.

Finally, 75 children with GDD (58 males and 17 females) aged 12 to 36 months were enrolled (Fig. 1). In the treatment group, the PGEE program was implemented from 45 children (35 males and 10 females), with an average age of 23.4 ± 7.6 months. No interventions, including the PGEE program, were applied to the 30 children in the control group (23 males and 7 females), with an average age of 24.5 ± 8.3 months. There were no significant differences in sex and age between the 2 groups (*P* > .05). This study was approved by the Ethics Committee of Yantai Yuhuangding Hospital and informed consent was obtained from the parents of each child. The clinical features of GDD for both groups have been presented in Table 1.

**Table 1:**
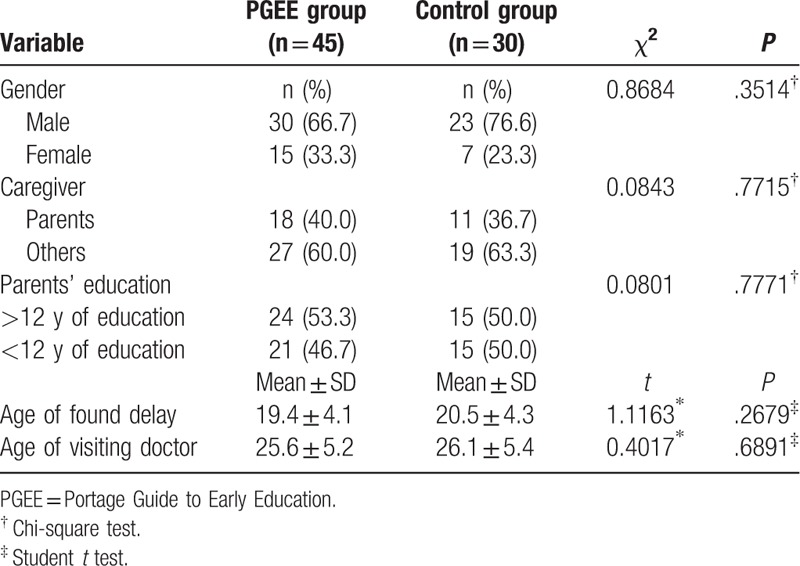
Basic characteristics of the participants.

### 2.2 Portage intervention

The PGEE program emphasizes the importance of the family and parents in the early intervention of GDD. The first training step is to understand the deficits in the child's ability and his/her learning interest and enthusiasm. A child's learning begins with an exploration of the environment. His/her positive attention to the surrounding environment suggests that their enthusiasm for learning is higher. If there is little interaction between the child and the external environment, it suggests that his/her interest in learning is low and that adults need to interact more with the child. The PGEE training plan includes different interesting games to promote the development of children's motor skills, language, intelligence, emotion, and sociality. The early experience of games plays an important role in determining the formation of neural circuits.

For children in the treatment group who were diagnosed with GDD, the PGEE program was performed to evaluate their abilities, defects, interests, and learning enthusiasm. The training program started with interesting and easy activities and gradually became more difficult. It was implemented by combining family and hospital intervention in a 1-to-1 ratio. The training program was performed once a week in the hospital, for 30 to 40 minutes, following which parent-guided family training was performed with specific training programs and instruments. One week after the training, the resulting effects were evaluated, the teaching plan was modified, and new goals were established. We developed individualized training programs according to each child's development level, and we taught parents the skills of playing games to improve children's development in a relaxing atmosphere. As therapists devoted more attention when the parents implemented the home activity program, parents’ adherence to the program was good. They performed the home activities for at least 2 hours per day.

### 2.3 Efficacy evaluation tools and methods

Developmental quotient (DQ) and social adaptability were measured before and 6 months after the implementation of the PGEE program in the treatment group. These parameters were also evaluated in the control group during the first visit and 6 months later.

Specialized workers made use of the Gesell Infant Development Scale (GESELL) (Chinese edition) to measure 5 parameters: gross motor skills (GM), fine motor skills (FM), adaptability (AD), language (L), and personal social activity (PS). The observed behavior pattern was evaluated according to the normal behavior. Infant development was assessed using the DQ, according to the following criteria: DQ < 70 abnormal, 70 to 84 suspected, and >85 normal. During treatment, DQ served as an indicator for degree of DD.

The Infants-Junior Middle School Students’ Social-Life Abilities Scales (SM scales) were used to measure social adaptability in children at different ages. The SM scales consist of 6 domains, including independent living, athletic abilities, operational abilities, communicative abilities, participation in collective activities, and self-management abilities. Children were classified as normal (SM score = 10) or as having a marginal defect (SM score = 9), mild defect (SM score = 8), moderate defect (SM score = 7), severe defect (SM score = 6), or extremely severe defect (SM score = 5). SM scales were applied before and after treatment.

### 2.4 Statistical analysis

Statistical analyses were performed with SPSS19.0 (IBM SPSS, Armonk, NY). This study included 2 groups, children with GDD who were provided PGEE intervention and those who were not. In order to compare the differences in the basic characteristics of the participants in these 2 groups, the Pearson Chi-square test was used to compare the proportions between 2 groups and the Student *t* test was used to compare 2 sample means when children were enrolled initially. Data were analyzed to determine the association between early intervention and developmental outcomes. The paired *t* test was used for comparing the scores on the pre-intervention and follow-up tests. The Student *t* test was used for comparing the mean differences in the scores on the developmental scales between the 2 groups. *P* < .05 was considered statistically significant and 95% confidence interval was used.

## 3 Results

We found 90 cases of children with GDD. Of these, 3 children aged below 1 year had hearing impairment, 2 had autism, and 1 had cerebral palsy. Three more who were aged over 3 years had been given early intervention previously, while 6 did not attend the PGEE program regularly. Therefore, these 15 cases were excluded from the present study. Finally, 75 children with GDD who met the inclusion criteria were recruited.

Participant characteristics, including gender, caregiver, parents’ education level, children's age at diagnosis with DD, and developmental age when they were brought to see a physician have been summarized in Table 1. Of the 75 children, 53 were boys (70.6%) and 22 were girls (29.4%). As evident from Table 1, more than half of the children with GDD were cared for by caregivers other than parents. Further, the DD of the children was generally noticed by their patients at a mean age of 17.77 ± 3.91 months, but they consulted a physician only by the mean age of 24.90 ± 6.43 months.

The Gesell and SM assessment scores of the 2 groups before intervention have been summarized in Table 2. Specifically, there were no significant differences between the PGEE group and the control group in the scores on the 5 Gesell developmental parameters (GM, FM, AD, L, and PS) and the SM total score (all *P*s > .05). In this study, most children with GDD had marginal (22 cases, 31.4%) mild (41 cases, 54.6%), or moderate defects in social ability (12 cases, %).

**Table 2:**
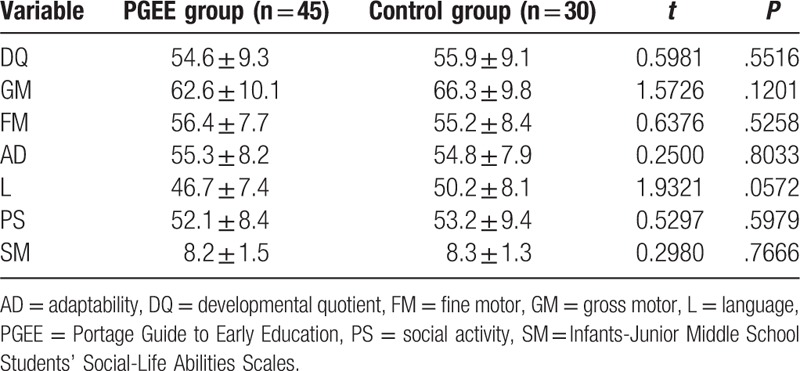
Comparison of Gesell and SM assessment scores between children in PGEE group and control group.

As summarized in Table 3, after the 6-month PGEE intervention, children's scores on the 5 developmental domains of Gesell and the SM total score increased as compared with their scores before the intervention (paired *t* test). This result showed that, after receiving early intervention for 6 months, on an average, the DQ of children in the treatment group increased by 10 points as compared with that before intervention.

**Table 3:**
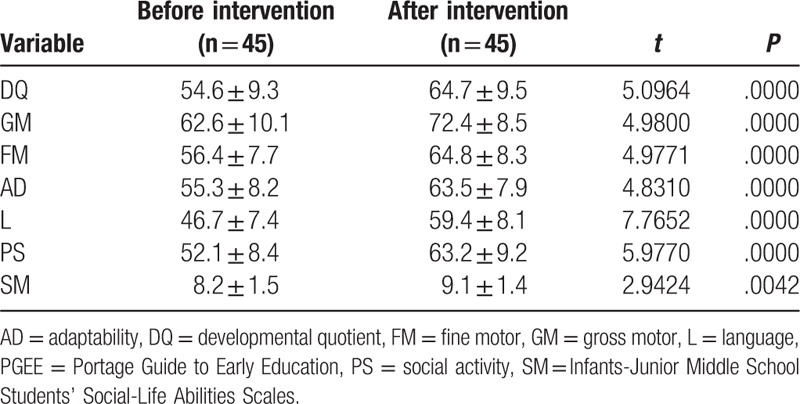
Comparison of Gesell and SM assessment scores in research group children before and after PGEE intervention.

The Student *t* test for the between-groups comparison showed that the 6-month PGEE intervention led to a significant increase in DQ values across 5 domains (GM, FM, AD, L, and PS) and in SM total scores in children with GDD (*P* < .05) (see Table 4). However, no significant improvements were observed in the control group after 6 months. Thus, without early intervention, children with GDD may not exhibit normal development by the natural course.

**Table 4:**
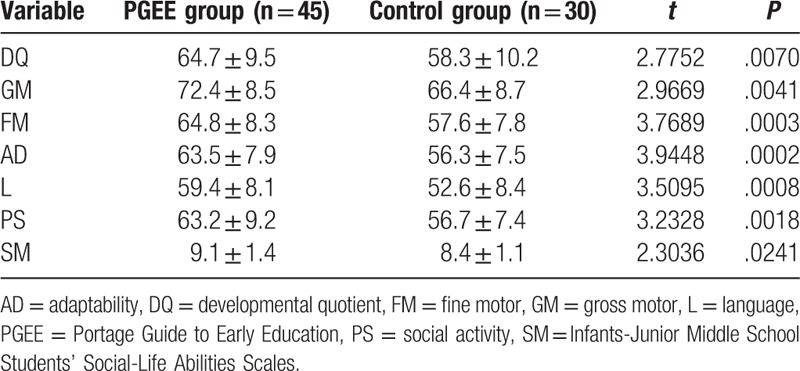
Comparison of Gesell and SM assessment scores between two groups after six-months PGEE intervention.

## 4 Discussion

Extensive DD in children can cause severe developmental disabilities. Although some delays in development may be temporary and may involve prognostic uncertainty, many are associated with neuropsychiatric disease. Some children with GDD may develop mental retardation or developmental disorders, and such observations demand early intervention.

### 4.1 Effects of early intervention on children with GDD

Early intervention includes a series of purposeful, organized, and enriched educational activities that promote the intellectual development of children. It is effective for the treatment of GDD and is based on the theory of brain plasticity,^[7]^ which describes how the structural and functional development of the human brain is shaped through contact with the external environment and interaction between genetic and acquired environments.^[8]^ The plasticity of the infant brain (0–3 years) is the greatest during cortical cell migration and vigorous glial cell proliferation. During this period, environmentally stimulated synaptic formations enhance the compensatory and adaptive functions of the brain, which include axon circuitous projections, unusual dendrite bifurcations, and unconventional synapses.^[9]^ Therefore, appropriate early intervention is critical for infants with GDD. However, in the present study, we found that a considerable number of parents did not consult a physician when they noticed a development delay in their child because they did not realize the adverse consequences of DD and the importance of early intervention. Such phenomena reflect that parents lack knowledge about children's cognitive development and that it is necessary to provide corresponding health education for parents.

The PGEE program is composed of 6 domains, including baby stimulation, motor skills, language development, cognitive ability, self-care, and social interaction. It is based on the concepts of family-training and individualized intervention, and focuses on cognitive and social adaptabilities to help children develop a variety of capabilities. The development of cognitive function is directly associated with the degree of functional recovery in children with GDD.^[10,11]^ In the present study, 6 months after the PGEE intervention, the Gesell assessment revealed significant improvements in the total DQ score as well as in the scores on the 5 DQ domains (GM, FM, AD, L, and PS), as compared with the corresponding scores before treatment and those in in the control group (*P* < .05). These findings suggest that PGEE-based early intervention can improve the DQ, cognitive function, and overall prognosis of children with GDD.

Intellectual and social adaptabilities develop most rapidly during infancy,^[12]^ and early intervention does not take into consideration the DQ and social adaptability of children with GDD.^[13]^ Our study revealed that most children with GDD had marginal or mild defects in social adaptability (66.17%). Parents tend to provide children with GDD more care, and often spoil and escort them, which causes such children to become more dependent and to exhibit poor living abilities, lack of capacity for self-management, and low social adaptability. In the present study, after the 6-month PGEE intervention, the SM scores in the treatment group were higher than those before treatment and those in the control group (*P* < .05). Our results suggest that PGEE intervention improves the social adaptability of children with GDD, lays a solid foundation for their future life, and reduces burdens on families and the society.

### 4.2 Roles of parents and families in the implementation of early intervention for children with GDD

It is advocated that early intervention should be provided in the natural environment, including learning and recreation environments, such as in family settings, child care centers, and the community.^[14]^ Specifically, the family provides the most important natural environment for children's development. The support provided by parents and caregivers is critical for the learning and emotional health of children, and family relationships create an environment in which parent–child interactions occur.^[15,16]^ An early intervention cooperative study (EICS) conducted an investigation on children and families who have participated in early interventions. It found that children with disabilities showed a more aggressive developmental trajectory when they lived in cohesive families, irrespective of the parents’ marital or socioeconomic status.^[17]^ Thus, family training is an important part of early intervention for children with GDD, and the attention of parents to family intervention, as well as their perceptions of intervention, have an irreplaceable role in the functional promotion and rehabilitation of children with GDD.^[18]^

The PGEE program attaches great importance to the role of parents in the early intervention for children with GDD and it recommends that parents conduct professional-guided early intervention in the family setting. Parental participation and execution plays a key role in the success of early intervention in children with GDD.^[19]^ One advantage of the PGEE program is individualized intervention, and in the present study, we developed individualized treatment and training methods based on the assessment of the developmental level, abilities, and specific needs of children with GDD. During early intervention, we encouraged parents to participate in the PGEE program and emphasized the “learn during play” principle to enhance the effectiveness of early intervention in children with GDD. Parents participated actively in this program and played the dual role of a parents and trainer. To maximize early intervention efficacy, it is important for parents to implement targeted education and training, based on the respective individualized treatment program for each child with GDD. In our study, the 6-month intervention using a weekly treatment program was sufficient for producing developmental benefits. Our results suggest that children diagnosed with GDD who receive early intervention will show improvements in developmental domains such as gross motor, fine motor, adaptability, language, and personal social activity. Further, the PGEE program provides more opportunities for training and practicing the therapeutic goals at home. This might explain why children in our study who participated in the PGEE program showed greater improvement.

In the present study, we could improve the development level of children with GDD through the PGEE intervention; however, there are still some limitations in our study. Although our result showed that the implementation of the PGEE program for at least 6 months can have positive effects, further research is needed to ascertain whether these effects will last and whether a longer duration of intervention is necessary. Another disadvantage is that our sample size was too small. Therefore, future studies need to employ a larger sample.

In summary, in the course of children's growth and development, DD affects the typical development process, especially in the maturation of the central nervous system. Therefore, early identification and effective early intervention are essential. The PGEE early intervention program was effective in improving the overall development and social adaptability of children with GDD. Therefore, it was concluded that the PGEE is an effective and feasible early intervention program that prevents and reduces GDD progression, thereby improving the prognosis and quality of life of children with GDD and their families.

## Author contributions

**Conceptualization:** Xiumei liu.

**Data curation:** Jing-Jing Ge, Xiu-Qing Dong.

**Formal analysis:** Xiu-Qing Dong.

**Investigation:** Jing-Jing Ge, Xiu-Qing Dong.

**Methodology:** Xue-Ming Wang, Jing-Jing Ge.

**Writing – original draft:** Xiumei liu, Xue-Ming Wang, Xiu-Qing Dong.

**Writing – review & editing:** Xue-Ming Wang.
